# Prevalence and associated risk factors for tuberculosis among people living with HIV in Nepal

**DOI:** 10.1371/journal.pone.0262720

**Published:** 2022-01-28

**Authors:** Nilaramba Adhikari, Ratna Bahadur Bhattarai, Rajendra Basnet, Lok Raj Joshi, Bhim Singh Tinkari, Anil Thapa, Basant Joshi

**Affiliations:** 1 Central Department of Public Health, Institute of Medicine, Tribhuvan University, Kathmandu, Nepal; 2 National Tuberculosis Control Center, Thimi, Bhaktapur, Nepal; 3 Department of Health Services, Ministry of Health and Population, Kathmandu, Nepal; 4 Institut de Recherche pour le Développement (IRD), UMR 1219, University of Bordeaux, Inserm, Bordeaux, France; Population Council, INDIA

## Abstract

**Background:**

Worldwide tuberculosis (TB) takes more lives than any other infectious diseases. WHO estimates around 68,000 incident TB cases in Nepal. However, in 2018 only around 27,232 new TB cases were reported in the national system, resulting around 40,768 incident TB cases missing every year in Nepal. National Tuberculosis Control Center carried out this study in anti-retroviral therapy (ART) sites to estimate the prevalence of TB and identify the associated risk factors for TB among the people living with Human Immunodeficiency Virus (PLHIVs) in Nepal.

**Methods:**

It was a cross-sectional institution-based study conducted between March and August 2018. Six ART sites with high caseloads of PLHIVs were selected. PLHIVs who were equal or above 18 years of age and were in ART program at the selected study sites were considered eligible for the study. Diagnosis of tuberculosis among PLHIVs who agreed to participate in the study was carried out as per the National Tuberculosis Management Guideline of National Tuberculosis Program of Nepal.

**Results:**

Among 403 PLHIVs, tuberculosis was diagnosed in 40 (9.9%) individuals. Median age of the participants was 36 (30–43) years. Prevalence of TB was significantly higher among male PLHIVs than female PLHIVs (13.6% Vs 5.8%; *P = 0*.*02*) and Dalit ethnic group compared to Brahmin/Chettri (22.0%Vs5.9%, *P = 0*.*01*). The risk of developing TB was found significant among those with HIV stage progressed to WHO stage 3 and 4 (OR = 4.85, *P<0*.*001*) and with the family history of TB (OR = 4.50, *P = 0*.*002*).

**Conclusions:**

Prevalence of TB among PLHIVs in Nepal was found 9.9%. Risk of developing TB was higher among PLHIVs who were male, Dalit, with HIV stage progressed to WHO stage 3 and 4 and with family history of TB. Hence, targeted interventions are needed to prevent the risk of developing TB among PLHIVs. Similarly, integrated, and comprehensive TB and HIV diagnosis and treatment services are needed for the management of TB/HIV co-infection in Nepal.

## Introduction

Worldwide tuberculosis (TB) takes more lives than any other infectious diseases [1]. For achieving the global tuberculosis control, early and accurate diagnosis of drug sensitive and drug resistant TB is crucial. However, underdiagnosis still remains as a pertaining obstacle especially in the countries where patient face substantial geographical and socio-economic challenges while accessing health care. Similarly, the scenario of many countries depicts that case detection relies on patients reporting symptoms to health care facility. In such condition of delays in case identification and management, there remains a higher probability of transmission and continuation of epidemic [2].

Transmission of tuberculosis depends on numerous factors, which includes the index case, the susceptibility of the exposed host, behaviour of bioaerosols, pathogen-inherent factors, and environment in which transmission occurs. Therefore, elimination of transmission relies on the ability to identify and treat the infected individuals at risk of becoming infectious, utilizing prognostic and diagnostic biomarkers to target preventive and curative therapies. Moreover, tuberculosis and HIV co-infection has fuelled the TB epidemic, presenting programmatic and treatment challenges across the world [3]. Co-morbidity of Human Immunodeficiency Virus (HIV) and tuberculosis, in the individual host, potentiate pathogens of one another, which eventually deteriorate the immunological functions. HIV coinfection is the prime risk factor for developing active TB in the high-burden settings. This in turn increases the susceptibility to primary infection or reinfection and the risk of TB reactivation for patients with latent TB. Similarly, infection of *Mycobacterium tuberculosis* also has a negative effect on the immune response to HIV, accelerating the progression from HIV infections to AIDS [4].

Globally HIV infection attributed 0.76 million incident TB cases along with an estimated 208,000 TB deaths among HIV positive people in 2019. Furthermore, the risk of developing TB among people living with HIV (PLHIVs) was 18 (range, 15–21) times higher than the general population [1]. Besides, estimation shows that one in every four deaths among PLHIVs was attributed to TB [5]. World Health Organization (WHO) estimates around 68,000 incident TB cases in Nepal. However, only around 27,232 new and relapse TB cases were reported in the national health management information system (HMIS) in 2019/20, resulting around 40,768 incident TB cases missing every year in Nepal [6]. Missing TB cases has posed significant challenges for the prevention, and control of TB in Nepal. Such undiagnosed TB cases has intensified transmission of TB in communities, putting vulnerable groups especially PLHIVs at higher risk of TB [7]. In 2019, the estimated number of PLHIVs in Nepal was 29,503 resulting adult (15–49 years) HIV prevalence of 0.13%. HIV epidemic in Nepal is concentrated largely among key population, notably among client of sex workers (9%), men who have sex with men and transgender (9%), male sex workers (3%), and people who inject drugs (3%). On the other hand, routine program data as of July 2020 shows only 19,410 PLHIVs on were anti-retroviral therapy (ART) along with 12% lost to follow up [8]. It reflects the urgent need to intensify the diagnosis of TB and HIV in health care settings to address the burden of HIV and TB co-morbidity in Nepal.

In the year 2004, WHO had published an interim policy on collaborative TB/HIV activities in response to demand from countries for immediate guidance on actions to decrease the dual burden of TB and HIV. This policy emphasized the need of collaboration between HIV programmes and TB control programmes to ensure access to integrated and quality-assured services for women, children, prisoners and for people who use drugs [9]. Later in the year 2009, National Tuberculosis Control Center (NTCC) in Nepal adopted TB/HIV strategy and policy with the aim to reduce the burden of TB and HIV in a population affected by both diseases in collaboration with National Center for AIDS and STD Control (NCASC). This strategy aimed to provide integrated and comprehensive TB and HIV prevention, treatment and care service as close as possible to detect more TB/HIV co-infected cases and prevent the case fatality rate from TB [10]. Despite the provision for TB screening among all PLHIVs in HIV care setting and HIV testing of all TB patients in TB care setting, yet the progress towards this indicator is very low. In 2019/20, only 51% of the TB patients were tested for HIV suggesting additional efforts to reach the target of 100% [6]. Besides, we could not find any published evidence suggesting the proportion of HIV patients who were screened for TB in HIV care setting in Nepal.

The information collected from health management information system (HMIS) in Nepal lacks validity and reliability due to incomplete, inconsistent, untimely recording and reporting along with lack of skilled manpower, necessary infrastructure, and tools in health care setting [11]. Hence the prevalence generated from the HMIS might not be reliable. Understanding the exact prevalence of TB among PLHIVs and the associated risk factors is crucial for effective planning and implementation of interventions for preventing TB and HIV co-infection in Nepal. In this scenario, NTCC carried out this study in ART sites with comparatively high caseloads to estimate the prevalence of TB and identify the associated risk factors for TB among PLHIVs in Nepal.

## Materials and methods

### Study setting

In Nepal, people who are diagnosed with HIV are enrolled in ART program irrespective of their CD4 cell count. There are 80 ART sites across the country to provide lifelong ART to PLHIVs [8]. The study was conducted at six ART sites namely BP Koirala Institute of Health Sciences (BPKIHS) in Sunsari, Tribhuvan University Teaching Hospital (TUTH) in Kathmandu, Sukraraj Tropical and Infectious Diseases Hospital in Kathmandu, Pokhara Academy of Health Sciences in Kaski, Bheri Provincial Hospital in Banke, and Seti Provincial Hospital in Kailali district of Nepal. These sites were selected based on the number of HIV patients registered for ART. These ART sites, with high caseload of PLHIVs on ART, were assumed to have greater population coverage representing all geographical regions and socio-demographic characteristics like gender, caste/ethnicity, religion, etc. Under HIV treatment and Care program, these sites have been serving as a referral treatment centers, providing advanced HIV treatment and care services. This study was conducted between March 2018 and August 2018.

### Study design

This was a cross-sectional institution-based study. PLHIVs with age 18 years and above and receiving ART at selected study sites were eligible to take part in the study. PLHIVs not meeting above inclusion criterions or PLHIVs who were already on TB treatment, PLHIVs of foreign nationality and PLHIVs showing unwillingness to participate in the study were excluded from the sampling frame. However, their right to know their infection status was protected through proper counselling and appropriate referral to testing sites. The sample size was calculated by using Epi-info STATCAL application assuming a two-sided confidence level at 95%, 80% power (1-B) of the study. The proportion of TB among PLHIVs was considered 8.5% for the sample size calculation, which is based on the finding from the similar sentinel surveillance conducted by NTCC on fiscal year 2013/14 [12]. The required sample size for the study was 405 assuming 5% non-response rate. The required number of sample size from each study site was selected proportionately using consecutive sampling method. During the entire study period, this study was able to include 403 PLHIVs.

### Data collection

Participants were informed about the voluntary participation in the study as well as assured about their right to leave study at any time for any reasons. Informed consent from each eligible PLHIV was obtained prior their participation in the study. Written consent was obtained from all eligible participants. Information obtained from all the eligible PLHIVs who went through screening and diagnosis for tuberculosis were kept confidential and secured at the respective study sites.

PLHIVs, at the study site, meeting the eligibility criteria were included in the study until the required sample size was reached. Based on the study objectives, a structured questionnaire was prepared and was finalized in consultation with technical experts of NTCC. Data variables namely gender, age, caste/ethnicity, religion, marital status, literacy status, occupation, risk group, family history of TB was collected from PLHIVs during interview prior sample collection while other covariates CD4 cell count, WHO stage, use of Isoniazid Preventive Therapy (IPT) were extracted from ART register. The outcome variable, TB status, was confirmed based on the results from sputum microscopy, chest X ray, Xpert MTB/RIF as well as by clinical diagnosis.

One day orientation was provided to health workers at ART sites (health workers, ART counsellors, study coordinators) prior to the study to enhance their capacity on proper counselling, collection and recording of the information in the study questionnaire, for screening and diagnosis of TB. Once the participants agreed to participate in the study, their socio-demographic information were collected through face-to-face interview using the structured study questionnaire. Then, they were screened for active TB based on presence of cardinal signs and symptoms (i.e., current fever, current cough, night sweat for more than 2 weeks, and weight loss of more than 3 kg in last 4 weeks), physical examination and Chest X ray indicative of tuberculosis. All the participants were further subjected for sputum microscopy. The two sputum samples from each respondent were taken, one spot sample on the same day and other morning sample on the next day. TB was confirmed if any one of the sputum samples (either spot or morning sample) in microscopy was found positive for *M*. *Tuberculosis*. All the samples with negative result from sputum microscopy were further subjected to Xpert MTB/RIF test. Patient whose sample was positive for *M*. *tuberculosis* in Xpert MTB/RIF test was considered as confirmed TB patient. Repeat Xpert MTB/RIF tests were conducted for sputum samples showing Rifampicin Resistance as per the testing guideline of National Tuberculosis Programme (NTP). In addition, national algorithm was followed for the clinical diagnosis of TB. All the diagnosed TB patients were referred to nearby DOTS clinics or drug-resistant tuberculosis (DR TB) treatment sites for TB treatment. Collected information by health workers were reviewed by study coordinators on daily basis to minimize possible errors in the study questionnaire.

### Data analysis

Data were entered directly into Statistical Package for Social Science (SPSS version 20) and analysed. Data entry and analysis were assigned to third party to minimize possible biases. Study focal person of NTCC crosschecked the entered data with the questionnaire and verified the validity of the entered data prior analysis.

Association between demographic (gender, age, caste/ethnicity, religion, marital status, literacy status, occupation, risk group, family history of TB) and clinical characteristics (CD4 cell count, WHO stage of HIV, IPT status) were explored with TB status using bivariate logistic regression analysis. P value of <0.05 was considered significant at 95% confidence interval for Odds Ratio (OR). Significant variables observed in bivariate analysis were further subjected to multivariate analysis to control for possible confounding and to assess the strength of association between different characteristics of PLHIVs and risk of developing TB. The fitness of regression model was tested by Hosmer and Lemeshow test. A multi-collinearity diagnostic test was applied between the independent variables before logistic regression was applied. Decisive criteria were set out to be a tolerance value of >0.1 or variance inflation factor (VIF) value of <10. All the variables were found to be within the criteria and were therefore used for logistic regression.

### Ethical approval

Ethical approval for this study was obtained from Nepal Health Research Council (NHRC), Nepal (Reg no. 379/2016). Permission from NTCC and ART sites were also obtained. Written informed consent was obtained from all study participants.

## Results

Among the total PLHIVs who were screened for TB, 40 were diagnosed with TB indicating 9.9% prevalence of TB among PLHIVs.

More than half participants (52.9%) in the study were male (Table 1). The median age of the participants was 36 (IQR, 30–43) years. Majority of the participants (41.4%) belonged to age group 30–39 years. In terms of their ethnicity, nearly half of the PLHIVs belonged to Janajatis (44.2%), followed by Brahmin/Chhetri (33.7%) and Dalits (14.6%) (Table 1).

**Table 1:** Prevalence of TB by socio demographic and clinical characteristics of PLHIVs (N = 403).

Characteristics of PLHIVs	Total (N)	%	Prevalence n (%)
**Gender**			
Male	213	52.9	29 (13.6)
Female	190	47.1	11 (5.8)
**Age group**			
Less than 20 years	8	2.0	1 (12.5)
(20–29) years	81	20.1	6 (7.4)
(30–39) years	167	41.4	18 (10.8)
(40–49) years	106	26.3	12 (11.3)
50 years and above	41	10.2	3 (7.3)
*Median Age (IQR)*	*36 (30–43) years*		
**Caste/Ethnicity**			
Dalit	59	14.6	13 (22.0)
Madhesi	13	3.2	1 (7.7)
Muslim	17	4.2	0 (0.0)
**Religion**			
Hindu	340	84.4	33 (9.7)
Buddhist	30	7.4	4 (13.3)
Islam	17	4.2	1 (5.9)
Christian	16	4.0	2 (12.5)
**Marital Status**			
Ever married	349	86.6	33 (9.5)
Unmarried	54	13.4	7 (13.0)
**Literacy Status**			
Illiterate	117	29.0	19 (16.2)
Literate	286	71.0	21 (7.3)
**Occupation**			
Agriculture	53	13.2	11 (20.8)
Labour	55	13.6	3 (5.5)
Housewife	114	28.3	10 (8.8)
Profession	66	16.4	5 (7.6)
Business	68	16.9	8 (11.8)
Others	47	11.7	3 (6.4)
**Family history of TB**			
Yes	43	10.7	10 (23.3)
No	360	89.3	30 (8.3)
**CD4 cell count**			
< 200 cells/UL	200	49.6	27 (13.5)
> = 200 cells/UL	203	50.4	13 (6.4)
**WHO Stage**			
Stage 3 and 4	103	25.6	24 (23.3)
Stage 1 and 2	300	74.4	16 (5.3)
**Ever used IPT**			
No	324	80.4	33 (10.2)
Yes	79	19.6	7 (8.9)
**Risk group**			
Injecting drug users (IDUs)	62	15.4	6 (9.7)
Client of FSW	83	20.6	11 (13.3)
FSW	13	3.2	1 (7.7)
MSM/TG	14	3.5	2 (14.3)
General Population	231	57.3	20 (8.7)

As presented in Table 2, prevalence of TB among PLHIVs was significantly higher among male than female (13.6% Vs 5.8%, P = 0.02) and among Dalit than Brahmin/Chettri ethnic group (22.0% Vs 5.9%, P = 0.01). The odds of developing TB among male PLHIVs was nearly three times (OR = 2.62) higher than female while Dalit PLHIVs were nearly four times (OR = 3.83) at higher risk of developing TB than Brahmin/Chettri. Among different age groups, the prevalence of TB was found highest among PLHIVs aged less than 20 years (12.5%) and lowest among aged 50 year and above (7.3%). However, the association was not found statistically significant. Among the other socio-demographic characteristics of PLHIVs, prevalence of TB was found comparatively higher among Buddhist (13.3%); among unmarried (13.0%); and among agricultural workers (20.8%). Importantly, none of the association were found significant. This study also assessed the risk of developing TB among PLHIVs by the different clinical characteristics. Among different clinical characteristics of PLHIVs, TB prevalence was found comparatively high among those PLHIVs with CD4 cell count less than 200 cells/UL (13.5%); whose HIV stage progressed to WHO stage 3 and 4 (23.3%); and who did not use IPT (10.2%). Prevalence of TB was found high among PLHIVs with family history of TB (23.3%); and who were clients of female sex workers (13.3%). The risk of developing TB was significantly higher among those with HIV stage progressed to WHO stage 3 and 4 (OR = 4.85, P<0.001) and among those with family history of TB (OR = 4.50, P = 0.002) (Table 2).

**Table 2:** Association between socio demographic and clinical characteristics with the development of TB in PLHIVs (N = 403).

Demographic characteristics	Prevalence n (%)	Unadjusted		Adjusted	
		OR (95% CI)	P value	OR (95% CI)	P value
**Gender**					
Male	29 (13.6)	2.56 (1.244–5.290)	0.01	2.62 (1.176–5.865)	0.02
Female	11 (5.8)	Ref			
**Age group**					
Less than 20 years	1 (12.5)	Ref			
(20–29) years	6 (7.4)	0.56 (0.059–5.336)	0.61		
(30–39) years	18 (10.8)	0.84 (0.098–7.271)	0.88		
(40–49) years	12 (11.3)	0.89 (0.101–7.903)	0.92		
50 years and above	3 (7.3)	0.55 (0.050–6.107)	0.63		
**Caste/Ethnicity**					
Brahmin/Chettri	8 (5.9)	Ref		Ref	
Janajati	18 (10.1)	1.80 (0.758–4.274)	0.18	1.85 (0.724–4.725)	0.20
Dalit	13 (22.0)	4.52 (1.761–11.610)	0.00	3.83 (1.344–10.916)	0.01
Madhesi/Muslim	1 (3.3)	0.552 (0.066–4.585)	0.58	0.64 (0.073–5.654)	0.69
**Religion**					
Hindu	33 (9.7)	Ref			
Buddhist	4 (13.3)	1.43 (0.471–4.353)	0.53		
Islam	1 (5.9)	0.58 (0.075–4.525)	0.60		
Christian	2 (12.5)	1.32 (0.289–6.104)	0.72		
**Marital Status**					
Ever married	33 (9.5)	0.70 (0.293–1.676)	0.43		
Unmarried	7 (13.0)	Ref			
**Literacy Status**					
Illiterate	19 (16.2)	2.44 (1.261–4.745)	0.01	2.21 (0.991–4.931)	0.05
Literate	21 (7.3)	Ref			
**Occupation**					
Agriculture	11 (20.8)	3.84 (1.001–14.741)	0.05		
Labour	3 (5.5)	0.84 (0.163–4.405)	0.84		
Housewife	10 (8.8)	1.41 (0.370–5.372)	0.61		
Profession	5 (7.6)	1.20 (0.273–5.297)	0.81		
Business	8 (11.8)	1.95 (0.491–7.795)	0.34		
Others	3 (6.4)	Ref			
**Family history of TB**					
Yes	10 (23.3)	3.33 (1.498–7.420)	0.003	4.50 (1.778–11.402)	0.002
No	30 (8.3)	Ref			
**CD4 cell count**					
< 200 cells/UL	27 (13.5)	2.28 (1.141–4.561)	0.02	1.46 (0.683–3.143)	0.33
> = 200 cells/UL	13 (6.4)	Ref			
**WHO Stage**					
Stage 3 and 4	24 (23.3)	5.39 (2.732–10.643)	0.00	4.85 (2.257–10.432)	0.00
Stage 1 and 2	16 (5.3)	Ref			
**Ever used IPT**					
No	33 (10.2)	1.16 (0.496–2.744)	0.72		
Yes	7 (8.9)	Ref			
**Risk group**					
Injecting drug users (IDUs)	6 (9.7)	1.13 (0.433–2.948)	0.80		
Client of FSW	11 (13.3)	1.61 (0.737–3.526)	0.23		
FSW, MSM /TG	3 (11.1)	1.31 (0.365–4.766)	0.67		
General Population	20 (8.7)	Ref			

## Discussion

Prevalence of TB among PLHIVs was found 9.9% which is slightly higher than the finding of similar study conducted by NTCC in fiscal year 2013/14 [12]. The prevalence was higher than the findings presented by other studies conducted at subnational level in Nepal [13,14]. It indicates that the prevalence of TB among PLHIVs is in increasing trend in Nepal.

In our study male PLHIVs were at greater risk of TB infection than female. This finding corresponds with similar studies conducted in different settings around the globe [15–21]. In developing countries where socio-cultural dynamics prefer male over female in terms of their movement and exposure [22], consequently exposure to TB bacilli could be high in male than female. Other reasons for this might be higher burden of HIV among male than female in general population [8]. Furthermore, burden of TB in the general population is also nearly twice among male than female [6]. A research finding shows more than a quarter of women (28%) in Nepal do not participate in decision making process resulting in low health seeking behaviour among female than male [23], it might have contributed for less number of TB diagnosis among female than male. In our study, higher risk of developing TB among Dalit PLHIVs than Brahmin/Chettri might be due to inequalities between them in accessing and utilizing health care services and resources. Evidence also show that Dalit in Nepal have been experiencing disparities in health outcomes and barriers for accessing desired health care services [24]. Significant proportion of Dalit are in lower wealth quintile, have poor households, and lack necessary infrastructure and resources as compared to non-Dalit ethnic group [25]. Inadequate nutrition and poor standards of living due to poor socio-economic status among Dalits might have increased the risk of developing TB than Brahmin/Chettri PLHIVs. This study found that PLHIVs with family history of TB were at more risk of developing TB. They were nearly five times more likely to develop TB than those without any family history of TB. PLHIVs having family members with TB could have increased their exposure to tuberculosis bacilli and have consequently increased the risk of developing TB. This finding resonates with other studies where they concluded household exposure to a known case of TB is an important risk factor for TB [26–29].

Age of PLHIVs was not found significantly associated with the risk of developing TB. This finding corresponds with other similar studies conducted in Brazil and Tanzania [30,31]. Illiterate PLHIVs were nearly three times more likely to develop TB than the literate. Although the association was not significant, other evidence has shown that having knowledge on measures of infection prevention and TB/HIV co-infection among PLHIVs reduced the risk of developing TB [32–35]. This study could not observe any association between occupation of the PLHIVs and the risk of developing TB. Contrary to our finding, few studies had reported increased risk of developing TB among people working at mines, silica dust, factories [36,37]. Although the burden of HIV is concentrated among key population in Nepal, this study could not establish any significant association between risk group of PLHIVs and the risk of developing TB. Contrastingly, a study conducted across Europe by Kruk A. found that IDUs were comparatively at higher risk of developing TB than others [38].

TB incidence was significantly associated with HIV stages of PLHIVs. PLHIVs with HIV stage progressed to WHO stage 3 and 4 were nearly five times at risk of developing TB than those in WHO stage 1 and 2. Generally, PLHIVs with HIV stage progressed to WHO stage 3 and 4 are more immunosuppressed and are more likely to get infected with different opportunistic infections [39,40]. It could be the reasons for the observed association. On the other hand, PLHIVs with CD4 cell count less than 200 cells per/UL were found to have increased risk of developing TB than those with CD4 cell count equal or greater than 200 cells per/UL. Although the association was not found significant on multivariate analysis, studies have proven that immunocompromised people are at more risk of developing TB. Even, latent TB infection progresses more rapidly to active TB disease with the suppressed immunity [41–44]. Despite proven effectiveness of IPT to reduce the risk of TB infection among PLHIVs and general population, this study could not observe any impact in the prevention of TB. In contrast to this study, other studies conducted in different geographical settings had found significant impact of IPT on preventing the risk of developing TB among PLHIVs [45–48].

The strength of this study is major ART sites with high caseloads of PLHIVs on ART were selected. Besides, these ART sites being referral centres, PLHIVs from different geographical areas are also covered in this study.

There are some limitations of this study. Important predictors for TB, like smoking habit, alcohol consumption, diabetic status, were missed in this study which could have otherwise explored additional evidence to strengthen TB/HIV co-infection program in Nepal.

## Conclusions

Prevalence of TB among PLHIVs was 9.9% in Nepal. The risk of developing TB among PLHIVs was significantly higher among male, Dalit ethnic group, those with HIV progressed to WHO stage 3 and 4 and among those with family history of TB. This study suggests that PLHIVs with certain characteristics are at greater risk of developing TB. Hence, targeted interventions are needed to minimize the risk TB among PLHIVs. Further studies are needed to explore the association between CD4 cell count, and effect of IPT with the risk of developing TB among PLHIVs. Finally, the implementation of integrated and comprehensive TB/HIV interventions is needed in health care settings to reduce the burden of TB and HIV co-infection through effective collaboration between National TB control program and HIV program in Nepal.

## Supporting information

S1 Table Results showing correlation between covariates and collinearity statistics. (DOCX)

S1 Dataset (XLSX)
