# Supplementary material for: Prevalence and associated risk factors for tuberculosis among people living with HIV in Nepal
Source: PLoS One. 2022 Jan 28;17(1):e0262720. doi: 10.1371/journal.pone.0262720 (PMC8797228; doi:10.1371/journal.pone.0262720)
Supplement: S1 Table — (DOCX) [file pone.0262720.s001.docx]

**Supplementary Table 1**

Results showing correlation between covariates and collinearity statistics

|  | **Correlation coefficient between covariates** | | | | | | **Collinearity Statistics** | |
| --- | --- | --- | --- | --- | --- | --- | --- | --- |
|  | Sex | Caste/Ethnicity | Literacy Status | WHO Stage | CD4 Cell Count | Family History of TB | Tolerance | VIF |
| Sex | 1 |  |  |  |  |  | 0.93 | 1.079 |
| Caste/Ethnicity | -0.028 | 1 |  |  |  |  | 0.96 | 1.045 |
| Literacy Status | -0.173 | -0.148 | 1 |  |  |  | 0.90 | 1.112 |
| WHO Stage | -0.12 | -0.062 | -0.127 | 1 |  |  | 0.95 | 1.053 |
| CD4 Cell Count | 0.132 | -0.087 | 0.109 | -0.101 | 1 |  | 0.95 | 1.049 |
| Family History of TB | 0.004 | -0.062 | -0.097 | 0.037 | 0.043 | 1 | 0.98 | 1.019 |
